# Evidence on point-of-care diagnostics for assessment of nutritional biochemical markers as an integral part of maternal services in low- and middle-income countries: systematic scoping review protocol

**DOI:** 10.1186/s13643-018-0932-1

**Published:** 2019-01-05

**Authors:** T. S. Dhlamini, D. Kuupiel, T. P. Mashamba-Thompson

**Affiliations:** Department of Public Health Medicine, School of Nursing and Public Health, University of KwaZulu-Natal, Durban, South Africa

**Keywords:** Point of care, Nutritional biomarkers, Maternal health, Low- and middle-income countries, Antenatal care

## Abstract

**Background:**

Malnutrition affects a third of the global population with low- and middle-income countries (LMICs) being the most affected. Most of those affected by malnutrition have limited means of determining their nutritional status. Recent developments of point-of-care (POC) diagnostics promise to enable early diagnosis of nutritional deficiencies or disease risk through biochemical indicator assessment. This provides potential opportunities for relatively simple interventions before the emergence of clinical symptoms. The main objective of this systematic scoping review is to map evidence on accessibility to POC diagnostic tests for the assessment of nutritional biochemical markers as an integral part of maternal health services in LMICs.

**Methods and analysis:**

We will search for relevant literature from the following databases: PubMed, Science Direct, EBSCOhost (Academic search complete, CINAHL with full text, MEDLINE with full text, MEDLINE), Google Scholar and World Health Organization library database. We will also search reference lists of included studies and existing networks such as organisations and conferences to source relevant literature. Primary research articles, published in peer-reviewed journals; review articles and grey literature that address the research question will be included. We will also search clinical trial registers to find relevant studies. Two independent reviewers will screen abstracts and full articles in parallel, from the included studies, using specific inclusion and exclusion criteria. Preferred Reporting Items for Systematic Reviews and Meta-Analyses (PRISMA) guidelines will be used for reporting the screening results. NVivo version 10 will be employed to enable content thematic analysis of the review findings. A narrative summary of the results will be presented according to the emerging themes.

**Discussion:**

We anticipate finding relevant literature on point-of-care diagnostic services for assessment of biochemical indicators as part of maternal services in low- and middle-income countries. The evidence obtained from the included studies when summarised will help to guide future research.

## Background

Malnutrition is a major health risk associated with increased healthcare-related costs estimated prevalence of 30–50% globally [1, 2]. In low- and middle-income countries (LMICs), maternal and child malnutrition includes undernutrition, overweight and obesity [3]. Studies have shown that globally, maternal undernutrition decreased in the past two decades as evidenced by low body mass index [3, 4]. However, maternal undernutrition continues to be prevalent in LMICs [3, 4]. It is reported that prevalence of maternal overweight has increased steadily since 1980 and exceeds that of underweight in all regions especially in LMICs [3, 4].

Maternal malnutrition increases the risk of poor pregnancy outcomes including obstructed labour, premature or low birth-weight babies and postpartum haemorrhage [5]. Postpartum haemorrhage has been associated with severe anaemia during pregnancy and increased mortality at labour [5]. Haemorrhage, hypertensive disorders and sepsis are responsible for more than half of maternal deaths globally with an estimated 99% in LMICs [6–8]. Also, more than a quarter of deaths were attributable to indirect causes including maternal malnutrition [9]. Interventions for diagnosing nutritional status at such as anthropometric indices based on body measurements, examination of clinical symptoms, dietary assessment and biochemical indicators (biomarkers) can help ensure early diagnosis and treatment of malnutrition. Nutritional biochemical markers are essential for determining nutrient exposure, status and effect of well-being [10].

Delayed disease diagnosis, as a consequence of poor access to laboratory infrastructure, has been demonstrated as one of the major problems in LMICs [11, 12]. The 2014 Amnesty International report on maternal health in South Africa highlighted the barriers such as poor access to healthcare including laboratory infrastructure resulting in pregnant women avoiding antenatal care, hence contributing to maternal deaths in the country [13]. Availability of accurate and reliable biomarkers in biological samples can enable early diseases diagnosis thus providing an opportunity for rapid clinical intervention [14]. Laboratory-based analysers such as ADVIA Centaur XP Immunoassay System (Siemens Healthcare GmbH), IMMULITE 2000 XPi Immunoassay System (Siemens Healthcare GmbH) and ACCESS 2 Immunoassay System (Beckman Coulter, Inc.) are available for quantification of nutritional biomarkers [14]. Nutritional assessments in traditional lab are expensive, time-consuming and can be even challenging with issues like fluctuating poor supply in economically poor settings with higher burden of malnutrition [14]. Laboratory-based nutritional diagnostics comes at a high cost and requires sample transportation and power supply [14]. These requirements pose a barrier to the accessibility of these diagnostics to resource-limited settings with the highest burden of malnutrition and poor access to laboratory infrastructure [14]. POC tests for nutritional biomarkers are cost-effective, are rapid and have been shown to produce reliable results [14]. With regards to disease diagnosis, time is of essence; POC tests can provide results much faster, typically less than an hour than the traditional laboratory approach.

A recent systematic review and meta-analysis provided evidence on the effectiveness of POC diagnostics on maternal health outcomes in HIV-infected women, globally [15]. Therefore, the main objective of this systematic scoping review is to identify and describe the scope and nature of the research evidence on accessibility to POC diagnostics for assessment of nutritional biochemical markers as an integral part of maternal services in low- and middle-income countries (LMICs). It is anticipated that the results of this study will inform policy makers, developers, and implementers of POC diagnostics for assessment of nutritional biochemical markers in LMICs.

## Methodology

The proposed scoping review will be guided by Arksey and O’Malley’s scoping review framework [16, 17] which has been further developed by Levac et al. and the 2015 Joanna Briggs Institute [18, 19]. Briefly, the framework involves the following steps: (i) identifying the research question, (ii) identifying relevant studies, (iii) selecting the studies, (iv) charting the data and (v) collating, summarising and reporting the results. We will also include the quality appraisal step to assess the quality of the included studies, as recommended by Levac et al. [18].

### Identifying the research question

The main research question for this systematic scoping review is as follows: What is the evidence on accessibility to POC diagnostic tests for assessment of nutritional biochemical markers as an integral part of maternal health services in LMICs?

The research sub-question is:

- What is the accessibility of POC diagnostic tests for nutritional biomarkers in maternal healthcare?

#### Eligibility of research question

The study will use an amended PICO (Population, Intervention, Comparison, Outcomes) framework to determine the eligibility of the research question (Table 1).

**Table 1:** Framework for determining eligibility of research questions

P	Maternal health patients in low- and middle-income countries
I	POC diagnostic services and nutritional status markers in maternal health
C	Any comparison used in primary studies on POC diagnostic services and nutritional status markers in maternal health
O	Accessibility to POC diagnostics for assessment of nutritional biochemical markers in LMICs

### Identify relevant studies

Primary research articles, published in peer-reviewed journals, review articles and grey literature that address the research question will be included in this study. All study designs will be included for review. We will search literature from the following databases: PubMed, Science Direct, EBSCOhost (Academic search complete, CINAHL and MEDLINE), Google Scholar and the World Health Organization library database. We will include searching clinical trials registers to find relevant ongoing studies. We will also search the reference lists of included studies and conferences websites to source relevant literature.

The search terms will include “nutritional,” “biomarkers,” “maternal health,” “point of care testing, point of care diagnostics, nutritional biomarkers, performance, and effectiveness”. MeSH (Medical Subject Headings) will be included in the search. Boolean terms, AND and OR, will be used to separate the keywords. We have conducted a pilot search using the above keywords to determine the feasibility of this study (Appendix). The search strategy will be adapted to each database. Each search will be documented in detail showing the keywords, date of search, search engine and number of publications retrieved.

### Study selection

#### Eligibility criteria

Guided by the study research question, we developed the inclusion/exclusion criteria to ensure correct identification and selection of relevant studies.

#### Inclusion criteria

To be included, studies must meet the following criteria:

- Primary studies published between 2008 and 2018
- Studies reporting evidence of POC testing for nutritional status biomarkers of maternal health services in LMICs
- Studies reporting evidence of screening and management of nutritional status biomarkers for maternal health services in LMICs

#### Exclusion criteria

We will exclude publications reporting the following:

- Evidence on POC diagnostics for other diseases other than nutritional status
- Evidence of POC diagnostics aimed at men and children
- Evidence of laboratory-based POC diagnostics
- Evidence from high-income countries

**Fig. 1:**
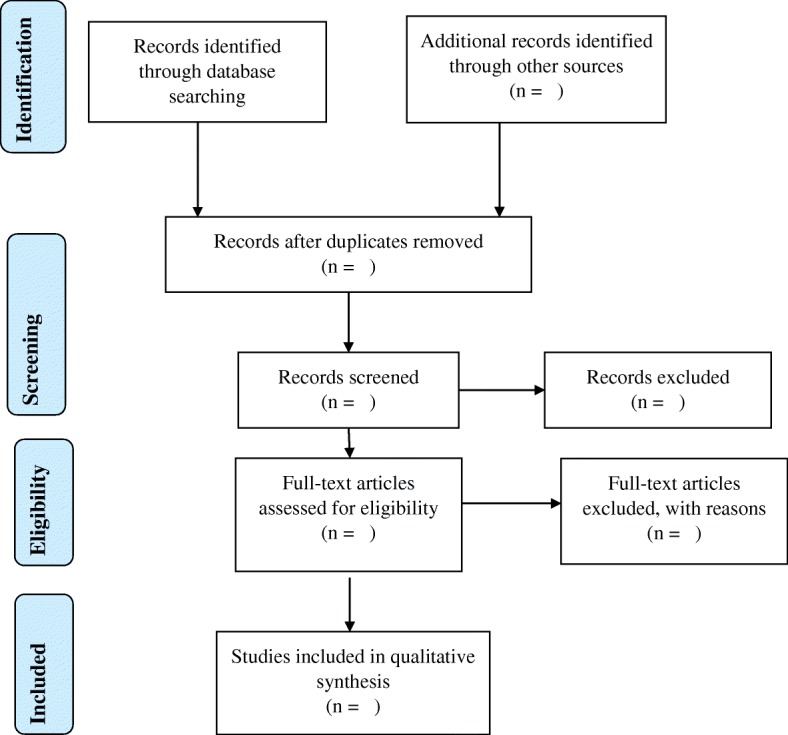
PRISMA 2009 flow diagram

This study will be conducted using a three-stage mapping strategy. The first stage will include comprehensive title screening which will be done by one reviewer. All eligible studies will be uploaded onto Endnote X7.4 software and duplicates removed. The final Endnote library will be shared among the review team. The second and third stages which will comprise of an abstract and full-article screening, respectively, will be conducted by two independent reviewers guided by the inclusion and exclusion criteria. In an event where a study/article could not be retrieved from the databases, we will request for the assistance from the University of KwaZulu-Natal library services and contact the authors to request for the article. Disagreement at the abstract screening stage will be resolve through discussions among the reviewers until consensus is reached. However, a third reviewer will be engaged to resolve disagreement at the full-article screening stage. Screening results will be reported using the adapted Preferred Reporting Items for Systematic Reviews and Meta-Analyses (PRISMA) guidelines [20] (Fig. 1).

### Charting the data

A data charting form will be developed and piloted (Table 2). The data charting form will be piloted by two independent reviewers using a random sample of 10 included studies for consistency. The data extraction form will then be modified as required based on feedback from the two reviewers. The data charting form will be constantly updated throughout the duration of the review. The NVivo computer software (version 11) shall be used to classify, sort, arrange, and examine relationships in the data and to extract the relevant outcomes and for the thematic analysis of the studies. The extracted data will be recorded in a data extraction form.

**Table 2:** Data charting form

Author and year	Study setting	Population	Intervention	Type of POC test	Country income classification	Aims	Study design	Sample size	Outcome measure	Key findings	Conclusion	Comment
ᅟ												

#### Collating, summarising and reporting of results

The primary aim of the study is to map evidence on the scope and effectiveness of POC diagnostics for nutritional biochemical indicators as an integral part of maternal health services in LMICs. The study will provide a narrative account of findings from existing literature through thematic content analysis of the extracted literature. The themes will be structured around the following interned outcomes: accessibility, effectiveness, availability of POC, nutritional status, screening of nutritional status and related health conditions. The themes will be coded in correspondence to the respective authors. NVIVO software version 13 would be used collectively to organise the data extracted from the included studies.

The below process will be followed:

- Coding
- Categorise codes into major themes
- Build theme-related themes (cut-and-paste technique)
- Display data
- Identify key patterns in the data and identify sub-themes
- Summarise

We will carefully interrogate the resulting themes and critically examine their relationship to the research question. Authors will also scrutinise the meanings of the findings as they relate to the overall aim of the study and address the implications for practice and for future research. We will analyse the resulting themes and critically examine their relationship to the research question. The reviewers will also analyse the meanings of the findings in relation to the aim of the study and the implications of these research, policy and practice.

#### Quality appraisal

We will include the quality appraisal step to assess the quality of the included studies, as recommended by Levac et al. [18]. The quality of the included studies will be assessed by use of the mixed method appraisal tool (MMAT) Version 2011 [21]. The MMAT allows the appraisal of most common types of study methodology and design [21]. This tool will enable us to examine the quality of the included studies such as the appropriateness of the aim of the study, adequacy and methodology, study design, participant recruitment, data collection, data analysis, presentation of findings, authors’ discussions and conclusions. The MMAT permits to concomitantly appraise and describe the methodological quality for three methodological domains: mixed, qualitative and quantitative (subdivided into three sub-domains: randomised controlled, nonrandomized and descriptive) [21].

## Discussion

The current scoping review will be conducted to provide evidence of the existence of POC diagnostic services for nutritional status markers in maternal services in LMICs. The Sustainable Development Goals (SDGs) focuses on increasing equitable coverage of quality health care and provision of integrated services as a means to reduce maternal mortality [22]. Reduction of maternal mortality has long been a global health priority and was a target in the UN Millennium Development Goals (MDG) framework and a key concern of the Global Strategy for Women’s and Children’s Health launched by the UN Secretary General in September, 2010 [23]. The goal was not reached by many LMICs in 2015. The World Health Organization (WHO) currently recommends nutritional biomarkers such as iron as part of essential antenatal testing [24].

The proposed systematic scoping review will include primary studies published from 2008 to 2018 and publications reporting evidence of POC testing for nutritional status biomarkers. Furthermore, publications reporting evidence of screening and management of nutritional status biomarkers and published and unpublished reports reporting on nutritional status biomarkers for maternal health services in LMICs will be included. The proposed systematic scoping review will exclude studies that report evidence of other POC diagnostics that are not aimed at screening or management of nutritional status in maternal health services. Evidence from high-income countries reported by studies, as well as, laboratory-based studies will also not be considered in this review. It will be essential to have a comprehensive and balanced account of previous work to provide a sound background of the research. Hence, this study will include evidence from articles that has been published from the past 10 years, 2008 to 2018.

It is anticipated that the results of the study will help identify requirement priorities for primary research in this area. The findings of the study may be of interest to stakeholders involved in the provision of maternal health services in LMICs. The findings of this study will be disseminated through peer-reviewed journal publications and conference presentations.

## Appendix

**Table 3:** Results of a pilot search in PubMed

Keywords search	Date of search	Search engine used	Number of publications retrieved
((((((((((“maternal health”[MeSH Terms] OR (“maternal”[All Fields] AND “health”[All Fields]) OR “maternal health”[All Fields]) OR (“prenatal care”[MeSH Terms] OR (“prenatal”[All Fields] AND “care”[All Fields]) OR “prenatal care”[All Fields] OR (“antenatal”[All Fields] AND “care”[All Fields]) OR “antenatal care”[All Fields])) OR (“pregnant women”[MeSH Terms] OR (“pregnant”[All Fields] AND “women”[All Fields]) OR “pregnant women”[All Fields])) OR (“prenatal care”[MeSH Terms] OR (“prenatal”[All Fields] AND “care”[All Fields]) OR “prenatal care”[All Fields])) AND (nutritional[All Fields] AND (“biomarkers”[MeSH Terms] OR “biomarkers”[All Fields] OR (“biochemical”[All Fields] AND “markers”[All Fields]) OR “biochemical markers”[All Fields]))) OR ((“point-of-care systems”[MeSH Terms] OR (“point-of-care”[All Fields] AND “systems”[All Fields]) OR “point-of-care systems”[All Fields] OR (“point”[All Fields] AND “care”[All Fields]) OR “point of care”[All Fields]) AND (“Diagnostics (Basel)”[Journal] OR “diagnostics”[All Fields]))) OR (“point-of-care testing”[MeSH Terms] OR (“point-of-care”[All Fields] AND “testing”[All Fields]) OR “point-of-care testing”[All Fields] OR (“point”[All Fields] AND “care”[All Fields] AND “testing”[All Fields]) OR “point of care testing”[All Fields])) OR (nutritional[All Fields] AND (“Markers”[Journal] OR “markers”[All Fields]))) AND accessibility[All Fields]) OR availability[All Fields]) AND (Low-and-Middle[All Fields] AND (“income”[MeSH Terms] OR “income”[All Fields]) AND Countries[All Fields])	10/11/2018	PubMed	580

## Abbreviations

LMICs: Low- and middle-income countries
MDGs: Millennium Development Goals
POC: Point of care
SDGs: Sustainable Development Goals
WHO: World Health Organization
